# Effect of Curcumin on Lifespan, Activity Pattern, Oxidative Stress, and Apoptosis in the Brains of Transgenic *Drosophila* Model of Parkinson's Disease

**DOI:** 10.1155/2014/606928

**Published:** 2014-04-17

**Authors:** Yasir Hasan Siddique, Falaq Naz, Smita Jyoti

**Affiliations:** Drosophila Transgenic Laboratory, Section of Genetics, Department of Zoology, Aligarh Muslim University, Aligarh, Uttar Pradesh 202002, India

## Abstract

*Background*. A time dependent loss of dopaminergic neurons and the formation of intracellular aggregates of alpha synuclein have been reported in PD model flies. *Methods*. The progeny (PD flies) expressing human alpha synuclein was exposed to 25, 50, and 100 *µ*M of curcumin mixed in the diet for 24 days. The effect of curcumin was studied on lifespan, activity pattern, oxidative stress, and apoptosis in the brains of PD model flies. The activity of PD model flies was monitored by using *Drosophila* activity monitors (DAMs). For the estimation of oxidative stress, lipid peroxidation and protein carbonyl content were estimated in the flies brains of each treated groups. The cell death in *Drosophila* brain was analyzed by isolating brains in Ringer's solution placing them in 70% ethanol and stained in acridine orange to calculate the gray scale values. *Results*. The exposure of flies to 25, 50, and 100 *µ*M of curcumin showed a dose dependent significant delay in the loss of activity pattern, reduction in the oxidative stress and apoptosis, and increase in the life span of PD model flies. *Conclusion*. Curcumin is potent in reducing PD symptoms.

## 1. Introduction

Parkinson's disease (PD) has been classified as a movement disorder and is characterized by the loss of dopaminergic neurons in substantia nigra [1]. The abnormal expression of alpha synuclein (*α*S) results in the formation of Lewy bodies: a pathological hallmark of PD [2]. The availability of various experimental models for PD, based on *α*S overexpression (mutant or wild form) in flies or mice, has led research scientists to study the effects of various compounds on the progression of PD symptoms [3]. Oxidative stress has been attributed as one of the important factors in progression of PD [4]. An emphasis has been given for the use of flavonoids to reduce the oxidative stress in the neurons [5–7]. Curcumin is the principal curcuminoid of the spice turmeric (*Curcuma longa*), a member of the ginger family [8]. Besides having a number of pharmacological properties [9], in our earlier study it was reported to delay the loss of climbing ability in the PD model flies [10]. In the present study, the effect of curcumin was studied on the life span, activity pattern, oxidative stress, and apoptosis in the brains of transgenic* Drosophila* model of PD.

## 2. Materials and Methods

### 2.1. *Drosophila* Stocks

Transgenic fly lines that expresses wild-type human synuclein (h-*α*S) under UAS control in neurons “w[*∗*];P{w[+mC] = UAS-Hsap/SNCA.F}”5B and GAL4“w[*∗*];P{w[+mC] = GAL4-elavL}”3 were obtained from Bloomington Drosophila Stock Center (Indiana University, Bloomington, IN). When the males of UAS (Upstream Activation Sequence)-Hsap/SNCA.F strains are crossed with the females of GAL4-elav. L (vice versa) the progeny will express the human alpha synuclein in the neurons [1].

### 2.2. Drosophila Culture and Crosses

The flies were cultured on standard* Drosophila* food containing 0.83% agar, 4.72% corn meal, 4.16% sugar, and 1.67% yeast at 25°C (24 ± 1) [11]. Crosses were set up as described in earlier published work [12]. The PD flies were exposed separately to different doses of curcumin (Sigma Aldrich, CAS 458-37-7) and mixed in culture medium at final concentration of 25, 50, and 100 *μ*M. The PD flies were also exposed to 10^−3^ M of L-dopamine. The UAS-Hsap/SNC.F acts as a control. The control flies were also separately exposed to the selected doses of curcumin.

### 2.3. Activity Pattern

From the 12th day onwards, the activity of flies (males) in all treated groups was analysed by using Drosophila Activity Monitor (TriTek, USA). The activity was recorded every hour for a total of 267 hrs and the data was analysed by Actogram J software. The results were presented as a chi-square periodogram [13, 14].

### 2.4. Lifespan Determination

For the determination of lifespan the newly enclosed male flies (control and PD) were placed in culture tubes (10 flies per tube) containing 25, 50, and 100 *μ*M of curcumin mixed in diet. The flies were transferred to new diet after every 3rd day and the number of dead flies were recorded at 3-day interval until the last one died [7].

### 2.5. Lipid Peroxidation Assay

Lipid peroxidation assay in the brain homogenate was performed according to the procedure described by Siddique et al. [15]. Reagent 1 (R1) was prepared by dissolving 0.064 g of 1-methyl-2-phenylindole into 30 mL of acetonitrile to which 10 mL of methanol was added to bring the volume to 40 mL. The preparation of 37% HCl served as the reagent R2. The brains of flies were isolated under stereo zoom microscope in ice cold Tris HCl (20 mM) (10 brains/group; five replicates/group). Homogenate was prepared in Tris HCl and centrifuged at 3000 g for 20 min and subsequently the supernatant was collected. In a microcentrifuge tube 1300 *μ*L of R1 was taken. A volume of 1 *μ*L (supernatant) was added along with 300 *μ*L of R2 vortexed and incubated at 45°C for 40 min. After incubation, the tubes were cooled in ice and centrifuged at 15,000 g for 10 min at 4°C and read at 586 nm.

### 2.6. Estimation of Protein Carbonyl Content

The protein carbonyl content was estimated according to the protocol described by Hawkins et al. [16]. The brain homogenate was diluted to a protein concentration of approx 1 mg/mL. About 250 *μ*L of each diluted homogenate was taken in eppendorf centrifuge tubes separately. To it 250 *μ*L of 10 mM 2,4-dinitrophenyl hydrazine (dissolved in 2.5 M HCl) was added, vortexed, and kept in dark for 20 min. About 125 *μ*L of 50% (w/v) trichloroacetic acid (TCA) was added, mixed thoroughly, and incubated at −20°C for 15 min. The tubes were then centrifuged at 4°C for 10 min at 9000 rpm. The supernatant was discarded and the pellet obtained was washed twice by ice cold ethanol : ethyl acetate (1 : 1). Finally, the pellets were redissolved in 1 mL of 6 M guanidine hydrochloride and the absorbance was read at 370 nm.

### 2.7. Analysis of Cell Death in* Drosophila* Brain

The cell death in* Drosophila* brain was analyzed as per the method described by Mitchell and Staveley [17]. Flies (5 flies/treatment; 5 replicates/group) were placed in 70% ethanol in a 2 mL microcentrifuge tube for a minute. The brains were isolated in Ringer's solution under stereo zoom microscope. After removing the Ringer's solution about 100 *μ*L of freshly prepared acridine orange (5 *μ*g/mL) was added for 5 minutes. The brain was rinsed by Ringer's solution, immediately viewed, and photographed through fluorescent microscope (Optika, Italy). The image analysis program Image J (available online at http://rsb.info.nih.gov/ij/) was used to analyze the gray scale values for each brain.

### 2.8. Statistical Analysis

The statistical analysis was done using Statistica Soft Inc. The mean values of various fly groups were statistically compared using Student's *t*-test.

## 3. Results 

**Figure 1:**
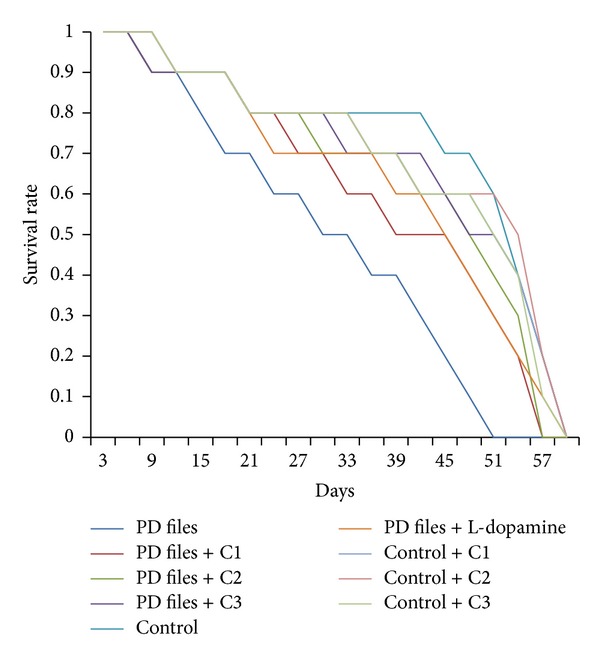
Effect of curcumin on survival rate measured in transgenic* Drosophila melanogaster *in various treated groups. (C1 = 25 *μ*M curcumin; C2 = 50 *μ*M curcumin; C3 = 100 *μ*M curcumin).

**Figure 2:**
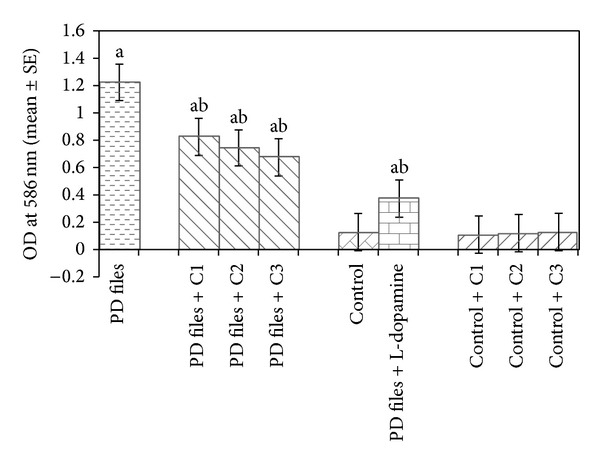
Effect of curcumin on lipid peroxidation measured in the brains of transgenic* Drosophila melanogaster *after 24 days of the exposure in treated groups. (C1 = 25 *μ*M curcumin; C2 = 50 *μ*M curcumin; C3 = 100 *μ*M curcumin; ^a^significant with respect to control, *P* < 0.05; ^b^significant with respect to PD model flies, *P* < 0.05).

**Figure 3:**
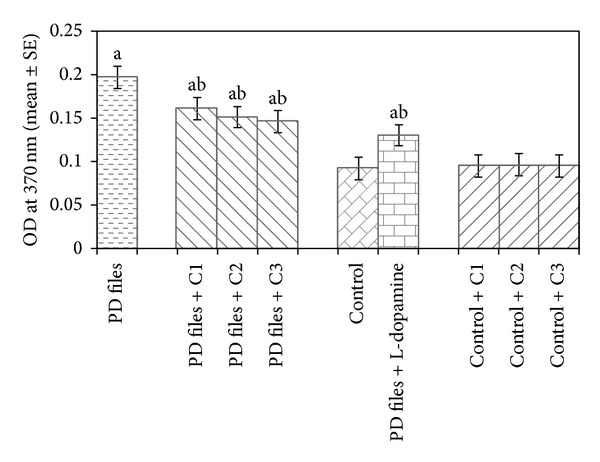
Effect of curcumin on protein carbonyl content measured in the brains of transgenic* Drosophila melanogaster *after 24 days of the exposure in various treated groups. (C1 = 25 *μ*M curcumin; C2 = 50 *μ*M curcumin; C3 = 100 *μ*M curcumin; ^a^significant with respect to control, *P* < 0.05; ^b^significant with respect to PD model flies, *P* < 0.05).

**Figure 4:**
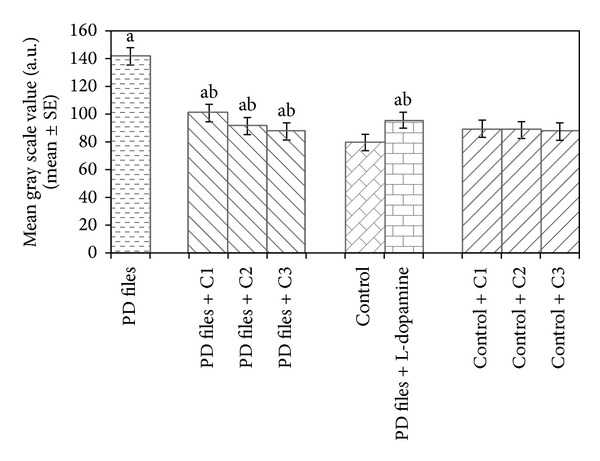
Effect of curcumin on mean gray scale value measured in the brains of transgenic* Drosophila melanogaster *after 24 days of the exposure in various treated groups. (C1 = 25 *μ*M curcumin; C2 = 50 *μ*M curcumin; C3 = 100 *μ*M curcumin; ^a^significant with respect to control, *P* < 0.05; ^b^significant with respect to PD model flies, *P* < 0.05).

The data collected for the male flies by* Drosophila* activity monitor (DAM) was analysed by chi-square periodogram. For control flies the number of peaks (significant) were more (Figures S1 (a) and (b)) compared to PD flies (Figures S2 (a) and (b)). A dose dependent significant delay in the loss of activity pattern was observed in the PD flies exposed to 25, 50, and 100 *μ*M of curcumin (Figures S3–5, (a) and (b)). No change in the activity pattern of control flies exposed to 25, 50, and 100 *μ*M of curcumin was observed (Figures S6–8, (a) and (b)). The PD flies exposed to 10^−3^ M of dopamine also showed a delay in the loss of activity (Figures S9 (a) and (b)) as compared to PD flies. The results obtained for the survival rate are shown in Figure 1. The survival rate was measured only in male flies. As is evident from Figure 1 the PD flies exposed to 25, 50, and 100 *μ*M of curcumin showed a dose dependent significant increase in the life span as compared to unexposed PD flies. The control flies showed a life span of about 60 days. The results obtained for the estimation of lipid peroxidation are shown in Figure 2. The PD flies exposed to 25, 50, and 100 *μ*M of curcumin showed a dose dependent significant decrease in the lipid peroxidation as compared to unexposed PD flies and control flies (Figure 2). The results obtained for protein carbonyl content are shown in Figure 3. A dose dependent significant decrease in the mean absorbance values was obtained in PD flies exposed to 25, 50, and 100 *μ*M of curcumin as compared to unexposed PD and control flies (Figure 3). The unexposed PD flies showed the highest mean absorbance value as compared to control flies (Figure 3). The results obtained for cell death in the brains of PD flies was calculated as mean gray scale values shown in Figure 4. A significant dose dependent decrease in the mean gray scale values was obtained for the PD flies exposed to 25, 50, and 100 *μ*M of curcumin as compared to unexposed PD and control flies (Figure 4). The PD flies exposed to 10^−3^ M of dopamine also showed a significant decrease in the mean gray scale value as compared to the unexposed PD flies and control flies (Figure 4).

## 4. Discussion

The results of the present study reveal that the exposure of PD flies to 25, 50, and 100 *μ*M of curcumin showed a dose dependent significant delay in the loss of activity pattern, reduction in lipid peroxidation, protein carbonyl content, apoptosis, and increase in the life span. Oxidative stress as a result of the accumulation of alpha synuclein has been reported in neurons of PD model flies [18]. It remains still unclear that the degenerating neuron itself or misfolded proteins directly causes toxicity during the progression of PD [19, 20]. In our earlier studies with the same fly models, various plant extracts and flavonoids have been reported to delay the loss of climbing activity and reduced oxidative stress [21–25]. Flavonoids have been reported to show improvements in cognition function possibly by protecting vulnerable neurons or by stimulating neuronal regeneration [26, 27]. In our present study, treatment of curcumin has shown reduction in lipid peroxidation and protein carbonyl content in the brains of PD model flies. This protection is attributed to an antioxidant nature of curcumin [28, 29]. Recent findings have suggested that flavonoids have a remodelling effect on the nature of *α*-synuclein fibrils, converting them into nontoxic, smaller amorphous aggregates, thus preventing the formation of reactive oxygen species [30]. On the other hand, an antioxidant nature of the curcumin is attributed to its unique conjugated structure that includes two methoxylated phenols [31]. It has been reported to inhibit the generation of reactive oxygen species (ROS) responsible for DNA and membrane damage [32]. Although the animals are well acquainted with the self-defense mechanism, an enhancement in stress beyond the capacity of an animal to cope up may result in cellular damage leading to the cell death [33]. The exposure of PD flies to curcumin showed a dose dependent decrease in the mean gray scale values, thus confirming an antiapoptotic activity of curcumin [34]. In earlier studies, curcumin has shown the neuroprotection in the 6-OHD model PD due to its antioxidant potential and its capability to penetrate into the brain [35]. It has been reported to alleviate *α*S-induced toxicity, reduce ROS level, and protect cell against apoptosis [36]. The aggregation of *α*S in the brain has been implicated as a crucial step in the formation of Lewy bodies and curcumin has antifibrillogenic and fibril-destabilizing properties, thus inhibiting the formation of alpha synuclein fibrils [37, 38]. In Drosophila curcumin have been reported to extend life span in a gender and genotype specific manner [39, 40]. In our present study, the life span and pattern activity were studied on male PD flies. There are reports on the life span extension of curcumin in mice [41] and* C. elegans *[42]. This extension is due to the neuroprotective and antiageing properties of curcumin [43]. The current pharmacotherapeutic approaches for PD involve improvement in striatal dopamine. The therapies involving natural antioxidants/plant products may be used as adjunct therapy [44]. The results obtained in our present study and our earlier study, in which the alginate-curcumin nanocomposite was studied using the same PD fly strain, results in neuroprotective effects [45]. Tetrahydrocurcumin has been reported to extend the life span of* Drosophila* and reduce the oxidative stress by regulating O-type forkhead domain transcription factor (FOXO) [46]. Despite having apoptotic properties in various cancerous cell lines there are reports of having antiapoptotic properties of curcumin that corroborate with the findings of our study [47–51]. Cancer cells can accumulate higher intercellular, cellular concentrations of vitamins/antioxidants than normal cells due to loss of homeostatic controls. The high concentration of the antioxidants can alter cancer cell metabolisms and cell signaling [52]. The present study was carried out using* Drosophila* as a model of PD expressing human wild type *α*-synuclein in neuron of fly and consequent locomotor dysfunction [1]. The present fly model mimics the motor impairments associated with PD and can be used to study whether or not a variety of compounds or drugs mixed in the fly culture medium have the neuroprotective potential [53].

## Supplementary Material

Chi-square periodogram for the control and treated groups.
