# Supplementary material for: Effect of Curcumin on Lifespan, Activity Pattern, Oxidative Stress, and Apoptosis in the Brains of Transgenic Drosophila Model of Parkinson's Disease
Source: Biomed Res Int. 2014 Apr 17;2014:606928. doi: 10.1155/2014/606928 (PMC4016861; doi:10.1155/2014/606928)
Supplement: Supplementary file 1 — Chi-square periodogram for the control and treated groups. [file 606928.f1.docx]

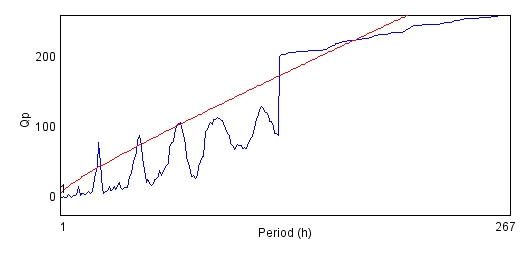

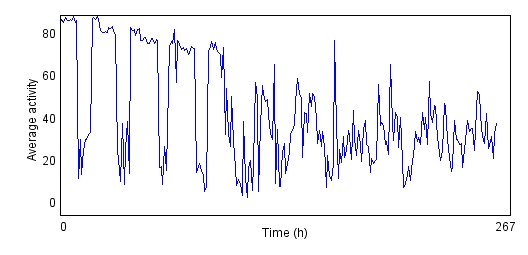
 (a) (b)


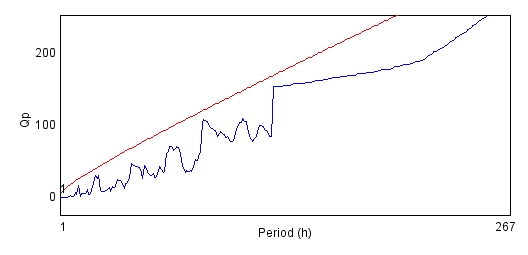
Fig. S1 (a) and (b) shows the average activity pattern and chi-square periodogram respectively for the control flies (N=20).


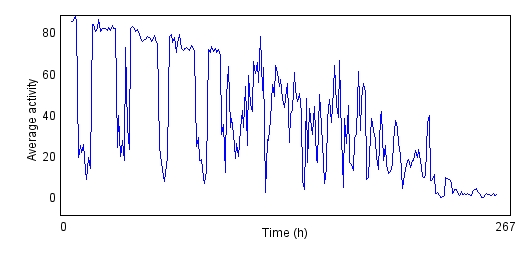
 (a) (b)


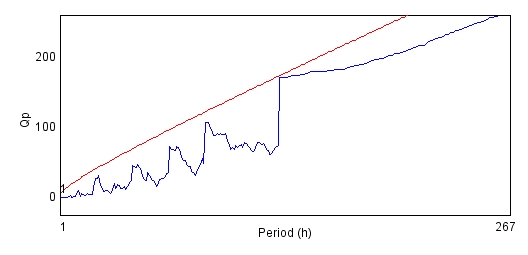
Fig. S2 (a) and (b) shows the average activity pattern and chi-square periodogram respectively for the PD flies (N=20).


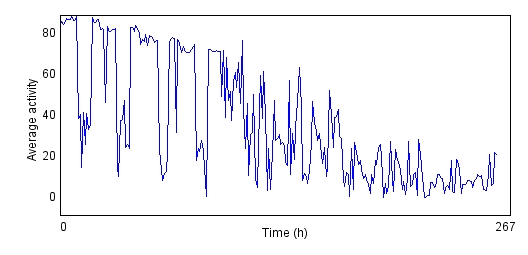
 (a) (b)

Fig. S3 (a) and (b) shows the average activity pattern and chi-square periodogram respectively for the PD flies exposed to 25µM of curcumin in diet (N=20).


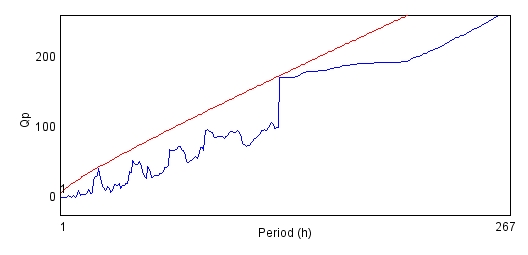

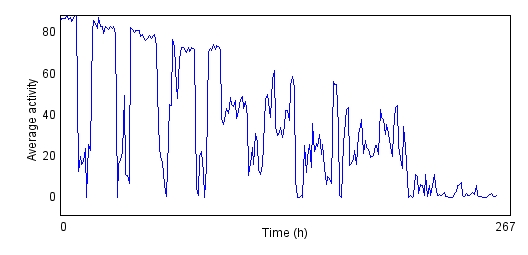


(a) (b)

Fig. S4 (a) and (b) shows the average activity pattern and chi-square periodogram respectively for the PD flies exposed to 50µM of curcumin in diet (N=20).


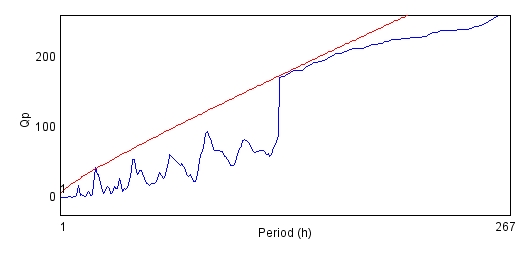

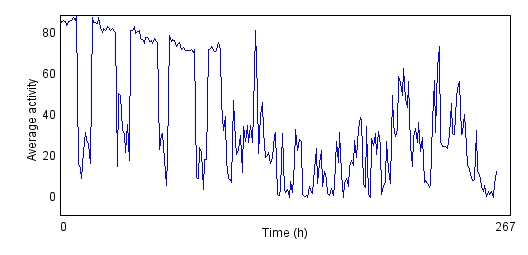


(a) (b)

Fig. S5 (a) and (b) shows the average activity pattern and chi-square periodogram respectively for the PD flies exposed to 100µM of curcumin in diet (N=20).


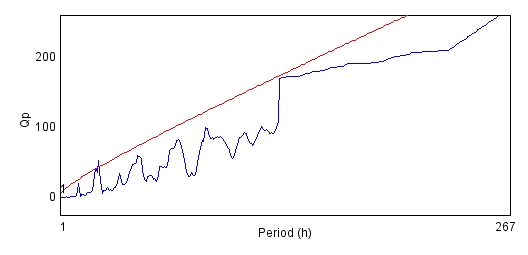

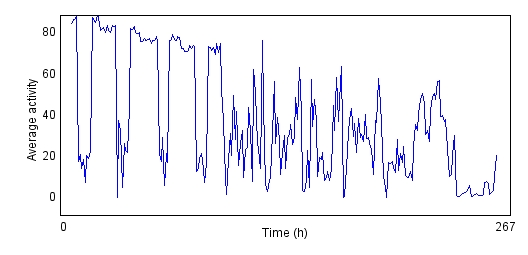


(a) (b)

Fig. S6 (a) and (b) shows the average activity pattern and chi-square periodogram respectively for the control flies exposed to 25µM of curcumin in diet (N=20).


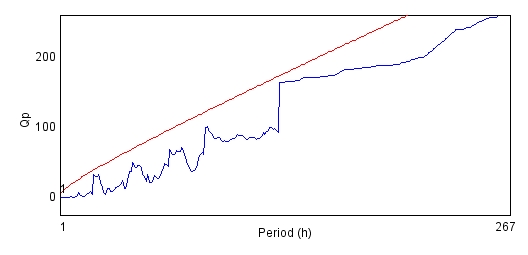

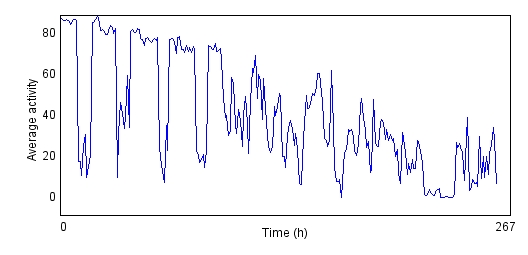


(a) (b)

Fig. S7 (a) and (b) shows the average activity pattern and chi-square periodogram respectively for the control flies exposed to 50µM of curcumin in diet (N=20).


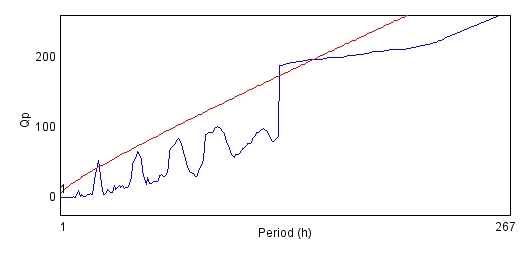

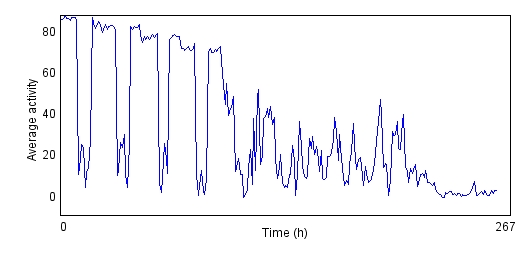


(a) (b)

Fig. S8 (a) and (b) shows the average activity pattern and chi-square periodogram respectively for the control flies exposed to 100µM of curcumin in diet (N=20).


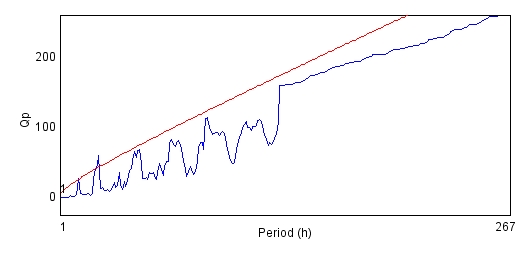

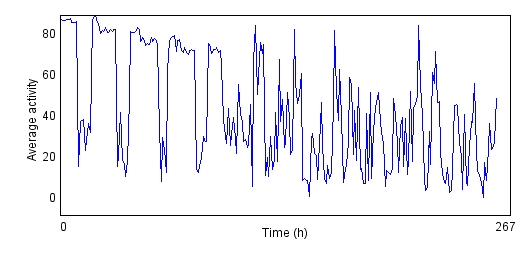
 (a) (b)

Fig. S9 (a) and (b) shows the average activity pattern and chi-square periodogram respectively for the PD flies exposed to 10^-3^µM of L-dopamine in diet (N=20).
